# Integration of transcriptome and DNA methylation reveals the mechanism of cilia-related genes in recurrent miscarriage

**DOI:** 10.1038/s41598-026-52154-x

**Published:** 2026-05-09

**Authors:** Jing Li, Junning Jing, Jingting Liu, Dengcai Zhang, Liyuan Zhang, Guangmei Xie

**Affiliations:** 1 Reproductive Medicine Center, Gansu Provincial Maternity and Child-Care Hospital, No. 999 Mogao Avenue, Anning District, Lanzhou City, 730070 Gansu Province China; 2 Laboratory Medicine Center, Lanzhou University Second Hospital, Lanzhou, Gansu Provincial China; 3 Department of Family Planning, Gansu Provincial Maternity and Child-Care Hospital, Lanzhou, Gansu Provincial China; 4 Clinical Laboratory Medicine Center, Gansu Provincial Maternity and Child-Care Hospital, Lanzhou, Gansu Provincial China; 5 Pathological Diagnosis Center, Gansu Provincial Maternity and Child-Care Hospital, Lanzhou, Gansu Provincial China

**Keywords:** Recurrent miscarriage, Cilia related genes, Methylation, Machine learning, Molecular regulatory network, Immunology, Biomarkers, Medical research

## Abstract

Recurrent miscarriage (RM), a complex pregnancy disorder with largely undefined molecular mechanisms, has been associated with epigenetic abnormalities in chorionic tissue. This study aimed to elucidate methylation-dependent cilia-related genes (CRGs) implicated in RM. An integrative analysis combining RNA sequencing, public transcriptome data, and DNA methylation profiles was conducted to identify RM-related CRGs. Machine learning algorithms were applied to determine the most relevant candidates. Immune infiltration profiling, gene set enrichment analysis (GSEA), and competitive endogenous RNA (ceRNA) network construction were employed to clarify molecular pathways. RT-qPCR validation was performed using clinical samples. Fourteen methylation-regulated CRGs were identified, among which SLC1A2 and ZDHHC20 were confirmed as key candidates. GSEA indicated their association with spliceosome, cell cycle, and proteasome pathways. Immune analysis demonstrated decreased infiltration of activated CD4^+^ T cells, effector memory CD4^+^ T cells, and Th2 cells in RM, with SLC1A2 and ZDHHC20 expression positively correlated with these immune subsets. The ceRNA networks indicated that SLC1A2 and ZDHHC20 were modulated by 7 miRNAs and 19 lncRNAs, respectively. RT-qPCR results showed significant overexpression of SLC1A2, but not ZDHHC20, in RM chorionic tissue. Collectively, SLC1A2 represents a methylation-regulated CRG that links ciliary impairment, immune imbalance, and epigenetic modulation in RM, revealing a novel molecular axis and suggesting its diagnostic and therapeutic potential.

**Supplementary Information:**

The online version contains supplementary material available at 10.1038/s41598-026-52154-x.

## Introduction

Recurrent miscarriage (RM), defined as two or more consecutive pregnancy losses, constitutes a major reproductive health concern, affecting approximately 1–5% of couples globally^1^. Epidemiological data indicate that RM imposes extensive repercussions beyond immediate physical complications such as hemorrhage and infection^2^. Evidence suggests that RM may predispose individuals to cardiovascular disorders and elevate the long-term risk of chronic diseases. Furthermore, RM has been linked to immune dysregulation, as repeated miscarriages can perturb immune homeostasis, predisposing affected women to autoimmune disorders including systemic lupus erythematosus and antiphospholipid syndrome^3–5^. The etiology of RM is multifactorial, including environmental exposures, genetic aberrations, anatomical anomalies, endocrine and immune dysfunctions, and infectious agents; nevertheless, nearly half of RM cases remain idiopathic^6,7^. Therefore, elucidating these associations and advancing diagnostic precision and individualized therapeutic strategies are essential for early identification of high-risk individuals and mitigation of patient burden.

DNA methylation, a conserved epigenetic modification involving cytosine methylation at CpG sites, modulates gene transcription, genomic imprinting, and cellular differentiation^8^. During early embryogenesis, methylation patterns undergo extensive reprogramming to establish totipotency and regulate lineage-specific transcriptional programs, and disturbances in this process have been associated with developmental abnormalities and pregnancy disorders^9,10^. Despite growing recognition of its biological relevance, investigations into aberrant DNA methylation mechanisms underlying RM remain limited.

Cilia are evolutionarily conserved, microtubule-based organelles that extend from the cell surface and function as essential hubs for cellular signaling and mechanosensation in most mammalian cells^11,12^. They are generally classified into motile cilia, which drive fluid movement (as in respiratory epithelia), and non-motile primary cilia, which serve as specialized sensory structures converting extracellular stimuli into intracellular responses^13^. Primary cilia have been recognized as central regulators of developmental signaling, tissue homeostasis, and disease mechanisms, particularly in organs undergoing intricate morphogenetic events^14,15^. The primary cilium, a single non-motile structure, is vital for coordinating signaling pathways fundamental to trophoblastic cell function. In human trophoblasts, it is indispensable for the modulation of cell invasion, an essential step in placental implantation and uterine spiral artery transformation^16,17^. Structural or signaling defects of primary cilia—such as disrupted Sonic Hedgehog (Shh) or Wnt signaling—impair trophoblastic migration, invasion potential, and endothelial remodeling, thereby contributing to pregnancy disorders including preeclampsia and RM^18,19^.

Mechanistically, the intraflagellar transport (IFT) system, particularly the IFT-B complex, is indispensable for the formation of cilia and the trafficking of signaling molecules. During the embryo implantation window, the IFT-B complex aggregates within primary cilia, and impaired energy metabolism has been associated with its mislocalization, leading to defective endometrial receptivity^20^. In addition to trophoblastic cells, primary cilia in decidual stromal cells regulate immune tolerance by maintaining the equilibrium of inflammatory cytokines; dysfunction of these cilia intensifies pro-inflammatory activity, disrupts maternal–fetal immune communication, and elevates the likelihood of miscarriage^21^. Collectively, primary cilia act as a central integrator linking cellular invasion, metabolic regulation, and immune modulation during early gestation. Clarifying the cilia-dependent molecular pathways underlying trophoblastic and decidual impairments may provide new therapeutic avenues for RM and other implantation-related disorders, contributing to the refinement of precision reproductive medicine.

This investigation utilized transcriptomic datasets derived from miscarriage tissues of RM patients and tissues from individuals undergoing elective abortion with normal pregnancies and no prior miscarriage history at our institution. Machine learning approaches were applied to identify RM-associated genes. Comprehensive analyses of the interactions between these key genes and the immune microenvironment were conducted to delineate their biological roles and regulatory networks. The objective was to elucidate the molecular mechanisms by which DNA methylation modulates cilia-related key genes implicated in RM.

## Materials and methods

### Sample collection and data source

Villous tissue samples were obtained from 8 patients with RM and 8 patients undergoing unplanned induced abortion (control) at Gansu Provincial Maternity and Child-Care Hospital between April and May 2023. Eligible participants in the RM group were women aged 20–40 years with at least two consecutive spontaneous abortions before 12 weeks of gestation. Controls consisted of women in early pregnancy electively terminating a pregnancy, with no history of spontaneous abortion, preterm delivery, diabetes mellitus, or hypertensive disorders of pregnancy, confirmed by ultrasound to have normal embryonic development and at least one prior successful live birth. Exclusion criteria for both groups comprised endocrine disorders (e.g., polycystic ovary syndrome, thyroid dysfunction), infections (e.g., Chlamydia trachomatis, Toxoplasma gondii), uterine structural anomalies (e.g., septum, fibroids), hereditary or acquired thrombophilias (e.g., factor V Leiden mutation, antiphospholipid syndrome), parental chromosomal defects, chronic systemic diseases (e.g., diabetes, hypertension, systemic lupus erythematosus), current or recent use of corticosteroids or immunosuppressants, active infectious diseases (e.g., hepatitis B/C, HIV), and embryonic chromosomal abnormalities detected by chorionic villus sampling or karyotyping. Maternal clinical data were recorded at the time of sample collection.

Clinical sample information for transcriptome are summarized in Table 1. In the RM group, the mean age was (32.0 ± 3.96) years, gestational age was (7.63 ± 0.74) weeks, and maternal BMI was (21.56 ± 1.17) kg/cm^2^. Correspondingly, the control group exhibited a mean age of (31.5 ± 3.70) years, gestational age of (7.00 ± 0.76) weeks, and maternal BMI of (20.39 ± 1.41) kg/cm^2^. Statistical analysis revealed no significant differences in age, gestational age, or BMI between the RM and control groups.

**Table 1:** Clinical sample information for transcriptome (n = 8 pairs).

Variables	Control	RM	Raw *P*-value	Adjusted *p*-value
	(n = 8)	(n = 8)		
Age, y	31.5 ± 3.70	32.0 ± 3.96	0.876	0.876
BMI, kg/m^2^	20.39 ± 1.41	21.56 ± 1.17	0.261	0.392
Gestational age, week	7.00 ± 0.76	7.63 ± 0.74	0.218	0.654

All collected samples were immediately flash-frozen in liquid nitrogen and preserved at − 80 °C until further analysis. Each specimen underwent whole-transcriptome sequencing and rigorous quality control (QC) to establish the RNA-sequencing (RNA-seq) dataset for subsequent investigation. Ethical approval for this research was granted by the Ethics Committee of Gansu Maternal and Child Health Hospital [(2024) GSFY Ethics Review [03] document], and written informed consent was obtained from all participants. The study adhered to the principles of the Declaration of Helsinki.

The dataset GSE198700 (GPL13534 platform, Illumina HumanMethylation450 BeadChip) was retrieved from the Gene Expression Omnibus (GEO) database (https://www.ncbi.nlm.nih.gov/geo/)^10^. It contained DNA methylation data from eight RM patients and eight individuals who underwent artificial abortions. Using “cilia” as the search term, gene sets were obtained from the MSigDB database (https://www.gsea-msigdb.org/gsea/msigdb/index.jsp), yielding 381 cilia-related genes (CRGs)^22^.

### RNA sequencing (RNA-seq) and data preprocessing

Total RNA was extracted from chorionic villus tissue samples of 8 RM patients and 8 controls using TRIzol reagent (Invitrogen, CA, USA). RNA concentration and integrity were assessed with a NanoDrop ND-1000 spectrophotometer (Wilmington, DE, USA) and a Bioanalyzer 2100 system (Agilent, CA, USA). Libraries were prepared with the Hieff NGS Ultifaillumima Dual-mode mRNA Library Prep Kit to obtain fragments of approximately 300 ± 50 bp. Sequencing was conducted on the Illumina NovaSeq 6000 platform using paired-end 150 bp reads (PE150 mode).

Raw sequencing reads in FASTQ format underwent quality control with fastp software (v 0.23.4)^23^, which removed adapter sequences, duplicates, and low-quality reads using default parameters. Clean reads were aligned to the human reference genome (Homo sapiens, GRCh38) with HISAT2 (v 2.2.1)^24^ (https://ccb.jhu.edu/software/hisat2), generating BAM files. In this process, the number of parallel threads was set to 8 via the parameter -p 8 to accelerate the alignment procedure. To optimize transcriptome analysis, the –dta option was applied. Considering the strand-specific nature of RNA sequencing data, the –rna-strandness RF parameter was used to specify the strand orientation. Additionally, based on the characteristics of the target genome, the maximum intron length was set to 50,000 using –max-intronlen 50,000. Transcripts were assembled and quantified in FPKM using the StringTie software (v 2.2.0)^25^ (https://ccb.jhu.edu/software/stringtie). The gene expression level was calculated using the following formula: FPKM = total exon fragments / [mapped reads (in millions) × exon length (in kilobases)]. During this step, specific parameters were configured: the number of parallel threads was set to 6 with -p 6; known gene annotation files were loaded via parameters such as -G reference_annotation.gtf to guide transcript assembly; the -e parameter was enabled to estimate gene expression levels; and the -B parameter was applied to generate BAM files required for subsequent analyses.

### Differential expression analysis in RM

We employed the DESeq2 package (v1.34.0)^26^ to identify differentially expressed genes (DEGs), differentially expressed microRNAs (DE-miRNAs), and differentially expressed long non-coding RNAs (DE-lncRNAs) from RNA-seq data comparing the RM group and the control group. The specific workflow included first constructing a DESeq2 object using the DESeqDataSetFromMatrix function, followed by dispersion estimation and negative binomial testing with the DESeq function. Differential expression results were extracted using the results function, with a filtering threshold set at |log₂ fold change (FC)|> 0.5 and a raw p-value < 0.05 (unadjusted) ; adjusted p-values (calculated via Benjamini–Hochberg method) for all differential molecules are provided in Supplementary Table S3 for statistical transparency. For the DNA methylation profiles of villus tissue from the GSE198700 dataset, comparing the RM group and the control group, we used the Limma package (v 3.54.0)^27^ to identify differentially methylated genes (DMGs). During the analysis, we fitted a linear model to the normalized data using the lmFit() function, accounting for the experimental design structure. The makeContrasts function was applied to define group comparisons, and the eBayes() function was used for empirical Bayes moderation of the standard errors to enhance the robustness of statistical inference. Finally, differential methylation sites were screened with the topTable function, applying a threshold of |log₂FC|> 0 and a raw *p*-value < 0.05. (unadjusted); adjusted p-values are detailed in Supplementary Table S4. Finally, ggplot2 (v 3.3.3)^28^ and pheatmap (v 1.0.12) (10.32614/CRAN.package.pheatmap) were employed to construct volcano and heat maps illustrating the expression patterns of DEGs, DE-miRNAs, and DE-lncRNAs.

To identify differentially expressed cilia-related genes (DE-CRGs) in RM, the CRG scores of RNA-seq samples were computed using the single-sample GSEA (ssGSEA) algorithm from the GSVA package (v1.42.0)^29^, with CRGs serving as the reference gene set. Samples were categorized into high- and low-score groups according to the median value and sample size. The DESeq^2^ package was then applied to detect DE-CRGs between these two groups, employing thresholds of |log_2_FC|> 0.5 and a raw *p* < 0.05 (unadjusted). Adjusted p-values are provided in Table S3 (Supplementary Materials).

### Identification of candidate genes, functional enrichment analysis, and construction of protein–protein interaction (PPI) network

Overlapping genes were identified between up-regulated DMGs and down-regulated DEGs, as well as between down-regulated DMGs and up-regulated DEGs. The merged set of overlapping genes was subsequently intersected with DE-CRGs to derive the candidate gene list. Functional enrichment of these genes during RM progression was analyzed through Gene Ontology (GO)^30^ and Kyoto Encyclopedia of Genes and Genomes (KEGG)^31^ analyses using the ClusterProfiler package (v4.6.0)^32^. GO terms were categorized into biological processes (BPs), cellular components (CCs), and molecular functions (MFs). Pathways with raw *p* < 0.05 (unadjusted) were retained and ranked in descending order. Likewise, significantly enriched signaling pathways (raw *p* < 0.05) (unadjusted) were obtained from KEGG analysis. To further delineate protein-level associations among the candidate genes, a PPI network was generated using the STRING database (http://string-db.org) with a confidence score threshold of > 0.40.

### Least absolute shrinkage and selection operator (LASSO) and support

Machine learning techniques were then applied to refine candidate genes derived from RNA-seq data. The glmnet package (v4.1–4)^33^ was used to perform LASSO regression, identifying feature genes with non-zero coefficients at the optimal lambda value. Subsequently, Support Vector Machine–Recursive Feature Elimination (SVM-RFE) was implemented using the e1071 package (v1.7.13)^34^ (https://CRAN.R-project.org/package=e1071), with performance evaluated through threefold cross-validation based on average error rates. The intersection of gene sets identified by both algorithms was visualized using the VennDiagram package (v1.6.20) (10.32614/CRAN.package.VennDiagram), yielding the final set of key genes.

### GSEA of key genes

To investigate the biological pathways associated with methylation and cilia-related key genes, GSEA was conducted using the clusterProfiler package. Correlation coefficients between each key gene and all other genes in the RNA-Seq dataset were first calculated, followed by ranking the genes in descending order based on their correlation strength. The msigdbr package (v7.5.1)^34^ was then employed to retrieve the CP: KEGG gene set as a reference for identifying enriched KEGG pathways within the tested gene set (raw *p* < 0.05) (unadjusted). Visualization of the top five enriched pathways corresponding to each key gene was performed with the enrichplot package (v1.18.4)^35^.

### Evaluation of immune cell infiltration in RM

Given the close connection between RM pathogenesis and immune system activity, the ssGSEA algorithm was applied to characterize the immune landscape of each sample in the RNA-seq dataset. The GSVA package (v1.42.0)^36^ quantified the relative abundance of 28 immune cell types across RM and control samples, and ggplot2 (v3.3.3) was used to generate stacked plots for graphical representation. Differences in immune cell infiltration between the two groups were statistically examined using the Wilcoxon rank-sum test (adjusted p < 0.05) . Spearman correlation analysis was further performed with the psych package (v2.3.9) (https://CRAN.R-project.org/package=psych) to determine associations between key genes and differentially infiltrated immune cells, considering correlations with |cor|> 0.3 and adjusted p < 0.05, to provide insights into potential immunoregulatory mechanisms. Adjusted p-values are provided in Table S8 (Supplementary Materials).

### Construction of competing endogenous RNAs (ceRNA) network based on key genes

To determine whether the key genes were under coordinated upstream regulation,

ceRNA networks were constructed by integrating DE-miRNAs and DE-lncRNAs from the dataset with the miRWalk (http://mirwalk.umm.uni-heidelberg.de/, v3.0, accessed on June 6, 2024) and miRNet (https://www.mirnet.ca/miRNet/Secure/DownloadView.xhtml, v3.0, accessed on June 6, 2024) databases. Initially, miRNAs interacting with the key genes were identified via miRWalk using a screening threshold of number_of_pairings > 20, followed by comparison with DE-miRNAs to select those exhibiting inverse expression trends relative to the key genes. Subsequently, miRNet was used to predict lncRNAs targeting these miRNAs. In this step, no confidence score cutoff was set, and all predicted interactions from the database were included. Finally, the intersection of the predicted lncRNAs and DE-lncRNAs was considered as the lncRNAs regulating the miRNAs. The resulting ceRNA network was visualized using Cytoscape (v3.7.2)^37^.

### Real-time quantitative polymerase chain reaction

Validation of the expression profiles of the identified key genes was conducted through quantitative real-time polymerase chain reaction (qRT-PCR) using ten additional pairs of clinical samples from the RM and control groups (Clinical sample information for qRT-PCR validation is provided in Table S1). Total messenger RNA (mRNA) was extracted with Trizol reagent (Ambion), and complementary DNA (cDNA) synthesis was performed using the SuperScript First Strand cDNA Synthesis Kit (Servicebio Company) following the manufacturer’s protocol. Amplification was carried out using the SYBR Green qRT-PCR kit on a CFX Connect Real-Time Quantitative Fluorescence PCR System (BIO-RAD). GAPDH cDNA served as the internal normalization reference. The primer sequences were as follows: 5′-TTTATTCGAGCTTCGGCGGG-3′ and 5′-ACTAGGTCACCCCCATCCTC-3′ (SLC1A2), 5′-CCACTGCTGGCTAGTTGGAA-3′ and 5′-AGCCACTTCTGGCATACTCA-3′ (ZDHHC20), and 5′-CGAAGGTGGAGTCAACGGATTT-3′ and 5′-ATGGGTGGAATCATATTGGAAC-3′ (GAPDH). Expression levels were quantified using the 2^–ΔΔCt^ method. Detailed sample information is provided in Table S2.

### Statistical analysis

All statistical analyses were performed using R software (v4.2.2). Inter-group comparisons for continuous variables (e.g., age, BMI, gene expression levels) were conducted using the Wilcoxon rank-sum test, with no multiple testing correction applied in primary analyses; Benjamini-Hochberg-corrected p-values correction applied for multiple comparisons to control the false discovery rate (FDR) are provided in Supplementary Tables (Table 1, S8) for reference. Associations between key genes and immune cell infiltration were evaluated using Spearman’s rank correlation coefficient, with no multiple testing correction applied; adjusted p-values are listed in Supplementary Table S8.

Differential expression/methylation analyses (e.g., identification of DEGs, DMGs, DE-CRGs): Raw p-values < 0.05 were used as the primary threshold for initial candidate screening (no multiple testing correction) to minimize false negatives—critical for retaining biologically relevant molecules (e.g., low-abundance cilia-related genes) that might be filtered out by strict FDR correction. Adjusted p-values (Benjamini–Hochberg method) are reported in Supplementary Tables S3–S5 to ensure statistical transparency.

GO/KEGG and PPI network construction: Raw *p*-values < 0.05 were used to retain broader biological information; adjusted p-values are detailed in Supplementary Table S7 to contextualize result robustness.

Statistical significance in primary analyses was defined as a raw p-value < 0.05 unless otherwise specified; adjusted p-values were only used for supplementary transparency and not for threshold-based screening.

## Results

### The identification of DEGs, DE-miRNAs, DE-lncRNAs, DMGs, and DE-CRGs was achieved

**Fig. 1:**
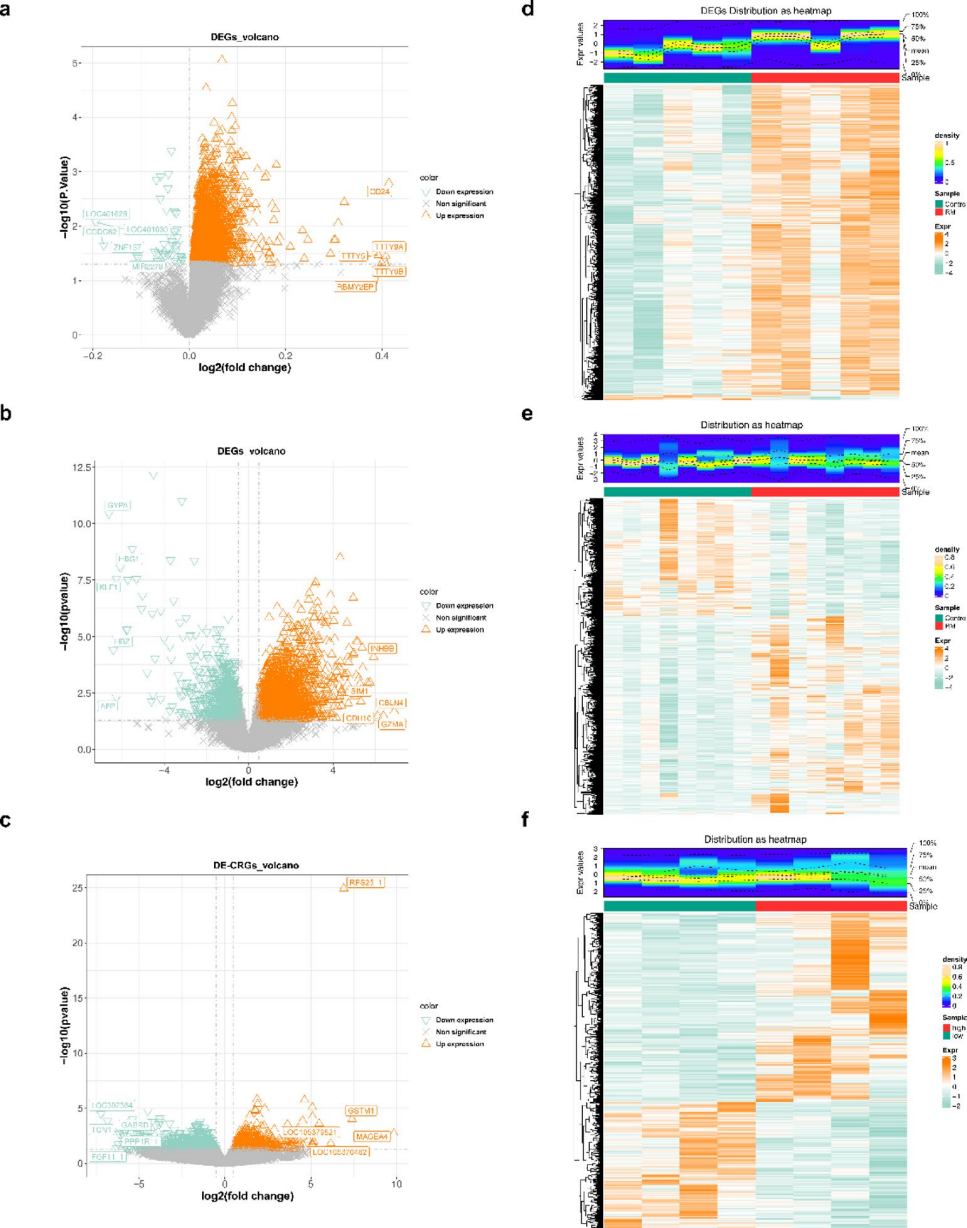
Identification of DEGs, DE-miRNAs, DE-lncRNAs, DMGs, and DE-CRGs between the RM and control groups. (**a-b**) Volcano plot (a) and heatmap (b) of differentially expressed genes (DEGs) between the RM and control groups. Screening thresholds: |log₂FC|> 0.5, raw *p*-value < 0.05. (**c–d**) Volcano plot (c) and heatmap (d) of differentially methylated genes (DMGs) between the RM and control groups. Screening thresholds: |log₂FC|> 0, raw *p*-value < 0.05. (**e–f**) Heatmap (e) and volcano plot (f) of differentially expressed cilia-related genes (DE-CRGs) identified after stratifying RM samples into high- and low-score groups based on cilia-related gene (CRG) scores. Screening thresholds: |log₂FC|> 0.5, raw *p*-value < 0.05. In the volcano plots, the x-axis represents log₂ fold change (log₂FC), and the y-axis represents − log₁₀ (raw p-value). In the heatmaps, orange indicates high expression, and green indicates low expression.

In the RNA-seq dataset, differential expression analysis between the RM and control groups identified 3,910 DEGs, comprising 2,536 upregulated and 1,374 downregulated genes (Fig. 1a–b). A total of 167 DE-miRNAs were detected, including 65 upregulated and 102 downregulated (Figure S1), along with 271 DE-lncRNAs, of which 187 were upregulated and 84 were downregulated (Figure S2). Detailed expression profiles of DEGs, DE-miRNAs, and DE-lncRNAs are presented in Table S3. Analysis of the GSE198700 DNA methylation dataset yielded 3,161 DMGs, with 187 upregulated and 84 downregulated (Fig. 1c–d), and their expression patterns are listed in Table S4. Based on the RNA-seq dataset, RM samples were stratified into high-score (n = 4) and low-score (n = 4) groups according to CRG scores, and subsequent differential analysis identified 1,295 DE-CRGs (Fig. 1e–f), with detailed expression profiles summarized in Table S5.

### Functional enrichment and PPI network of candidate genes

**Fig. 2:**
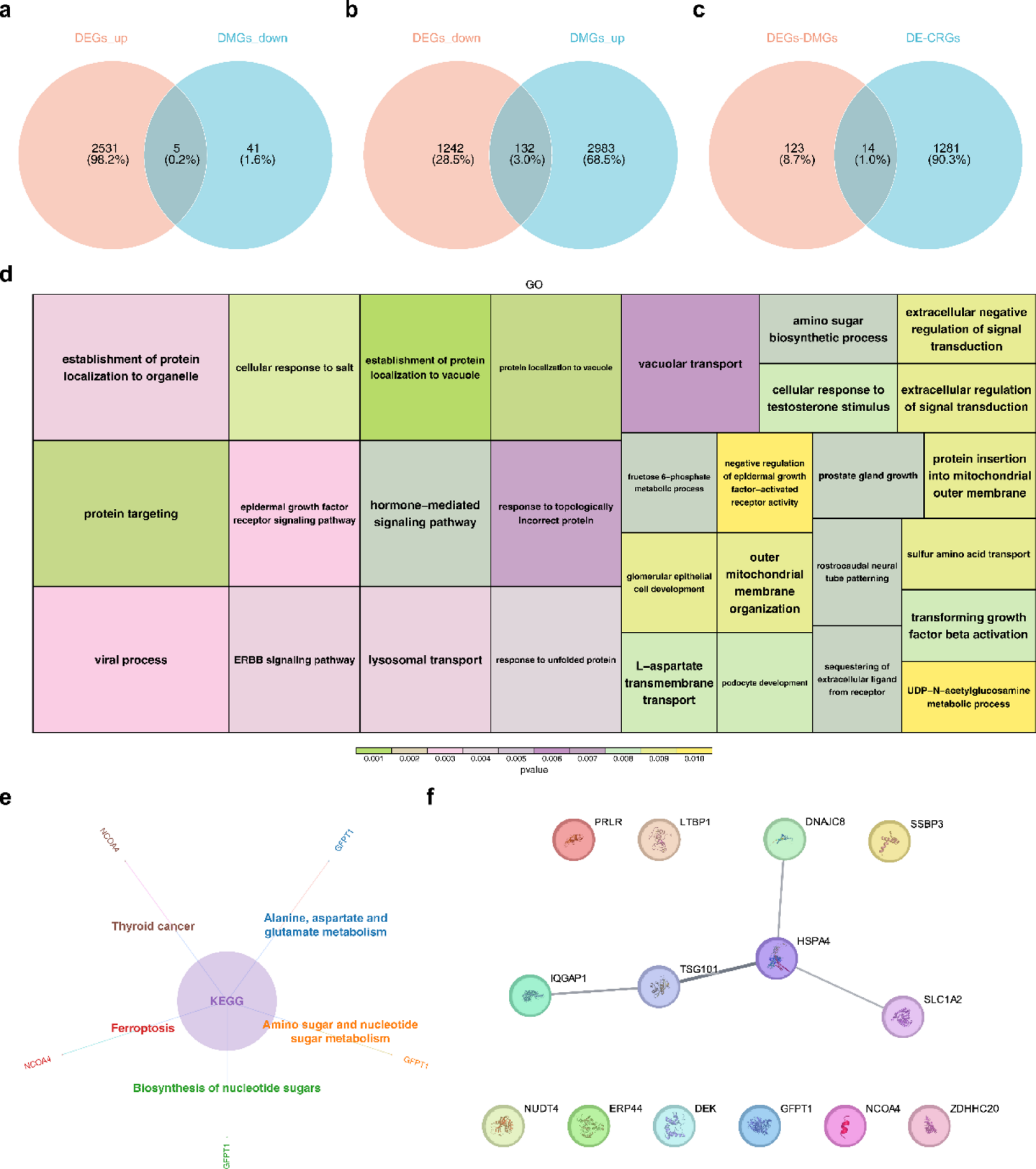
Identification, functional enrichment, and PPI network of candidate genes. (**a**) Overlapping genes between downregulated DMGs and upregulated DEGs. (**b**) Overlapping genes between upregulated DMGs and downregulated DEGs. (**c**) Shared genes obtained from the intersections in Figs. 2a, b combined with DE-CRGs, defined as candidate genes. (**d**) GO enrichment analysis of 14 candidate genes, highlighting the top 30 enriched GO terms.In the figure, green to yellow represent the significance range. Different color modules represent different levels of significance. Among them, green has the strongest significance with *p* < 0.001, while yellow has relatively weaker significance with *p* < 0.01, but it still has significance. (**e**) KEGG analysis identifies five pathways jointly enriched among the 14 candidate genes. (**f**) PPI network of the 14 candidate genes, showing five proteins with mutual interactions and nine isolated proteins; line width reflects interaction strength.

Through integrative analysis, we identified 14 cilia-related candidate genes regulated by methylation (Fig. 2a-c). The screening strategy was based on the negative regulatory relationship between DNA methylation and gene expression: first, genes with decreased methylation levels and increased expression levels were selected (Fig. 2a, intersection of downregulated DMGs and upregulated DEGs), which may be activated due to hypomethylation. Meanwhile, genes with increased methylation levels and decreased expression levels were also selected (Fig. 2b, intersection of upregulated DMGs and downregulated DEGs), which may be suppressed due to hypermethylation. These two sets of genes were merged and then intersected with DE-CRGs, ultimately yielding 14 candidate genes (Fig. 2c), including DEK, DNAJC8, ERP44, GFPT1, HSPA4, IQGAP1, LTBP1, NCOA4, NUDT4, PRLR, SLC1A2, SSBP3, TSG101, and ZDHHC20. Detailed information on overlapping genes is presented in Table S6. Functional annotation revealed that these genes were enriched in 235 GO terms (Fig. 2d, Table S7), comprising 19 cellular components, 37 molecular functions, and 179 biological processes. The most significant GO terms involved protein localization to vacuoles, protein targeting, transcription coactivator activity, and the endoplasmic reticulum–Golgi intermediate compartment. KEGG pathway analysis indicated that the candidate genes were mainly implicated in alanine, aspartate, and glutamate metabolism; nucleotide sugar biosynthesis; thyroid cancer; and ferroptosis (Fig. 2e, Table S7). These findings suggest that the identified genes participate in amino acid synthesis, degradation, and interconversion, contributing to the regulation of cellular metabolic balance. In the PPI network constructed from the 14 candidate genes, 14 nodes and 4 interaction pairs were observed. HSPA4 interacted with DNAJC8, TSG101, and SLC1A2, while TSG101 was connected to HSPA4 and IQGAP1 (Fig. 2f).

### Identification of key genes and KEGG pathway enrichment

**Fig. 3:**
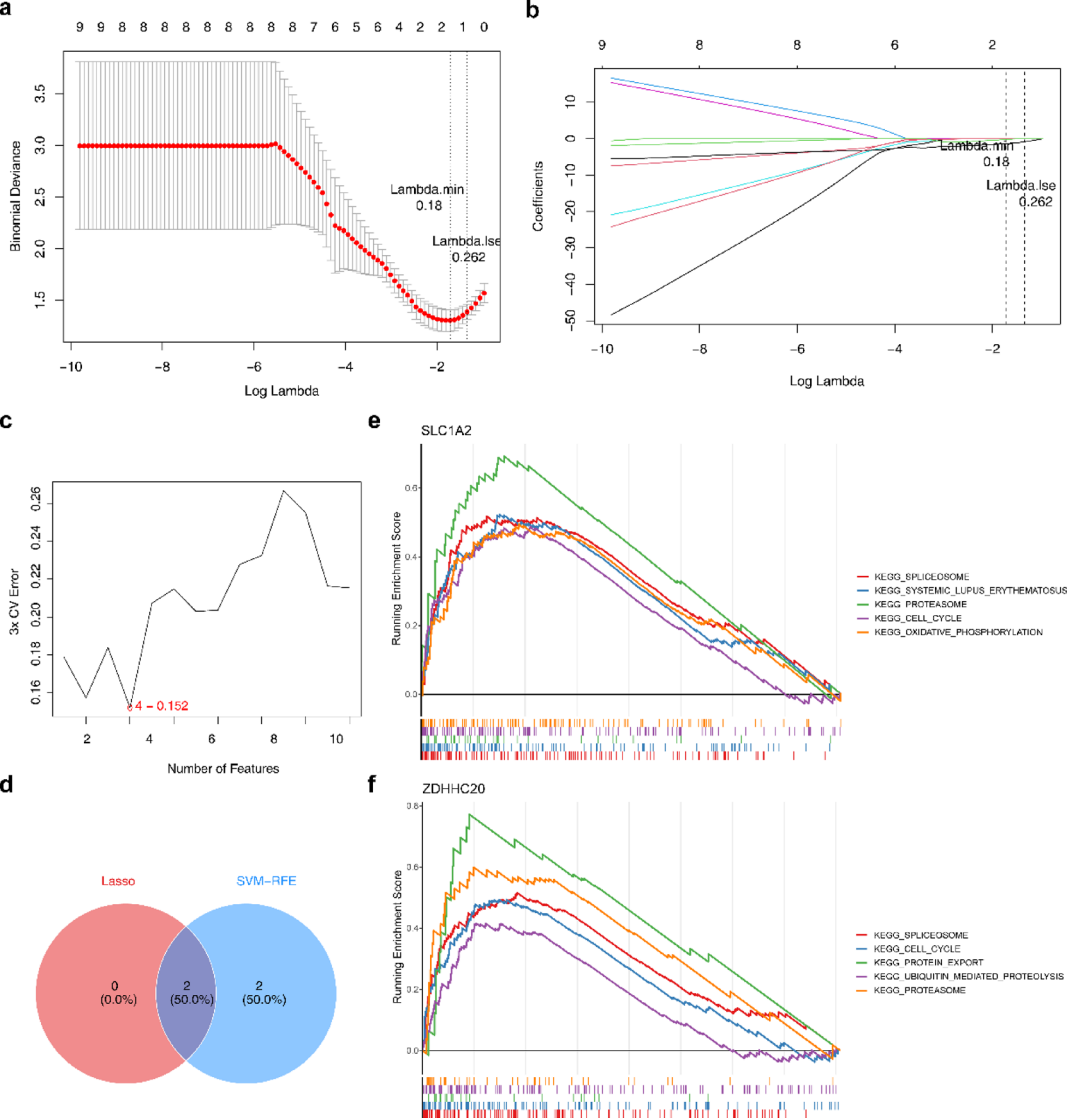
Identification of key genes and Gene Set Enrichment Analysis (GSEA). (**a**) LASSO coefficient path plot. The x-axis (Log Lambda) denotes the logarithmic transformation of λ, while the y-axis (Binomial Deviance) indicates model deviance. (**b**) Cross-validation (CV) error curve of LASSO regression. The x-axis (Log Lambda) represents the logarithmic value of λ, and the y-axis (Cross-Validation Error) corresponds to the prediction error obtained through CV. Optimal λ values (Lambda.min or Lambda.1se) were selected to determine DNA methylation–regulated cilia-related key genes. (**c**) Key genes associated with RM identified using the SVM-RFE algorithm. The x-axis (Number of Features) shows the number of genes incrementally included in model training (ranging from 2 to 14), and the y-axis (3CV MSE) represents the mean squared error from threefold cross-validation, quantifying the difference between predicted and observed values. The lowest MSE was achieved when four features were included, indicating optimal model performance. (**d**) Venn diagram illustrating the overlap of key genes identified independently by the LASSO and SVM-RFE models. (**e**–**f**) GSEA plots depicting the KEGG pathway enrichment of SLC1A2 and ZDHHC20. The top five KEGG pathways with the lowest P-values for each gene are displayed separately.

To identify cilia-associated genes modulated by DNA methylation in RM, two machine learning approaches were applied to refine the 14 initial candidates. The LASSO algorithm, using a minimum lambda value of 0.18, selected two genes—SLC1A2 and ZDHHC20—from the candidate pool based on non-zero coefficients (Fig. 3a–b). In parallel, the SVM-RFE method identified four optimal genes (ZDHHC20, SSBP3, SLC1A2, and DEK) relevant to RM (Fig. 3c). Intersection analysis of the two models (Fig. 3d) confirmed SLC1A2 and ZDHHC20 as the final key genes for subsequent analyses. GSEA demonstrated that SLC1A2 participated in 44 enriched pathways (Fig. 3e), such as systemic lupus erythematosus, cell cycle, and oxidative phosphorylation, while ZDHHC20 was involved in 38 pathways (Fig. 3f), including cell cycle, protein export, ubiquitin-mediated proteolysis, with spliceosome and proteasome emerging as shared enrichment pathways for both genes.

### The ssGSEA was used to evaluate the immune cell conditions in the control and RM groups.

**Fig. 4:**
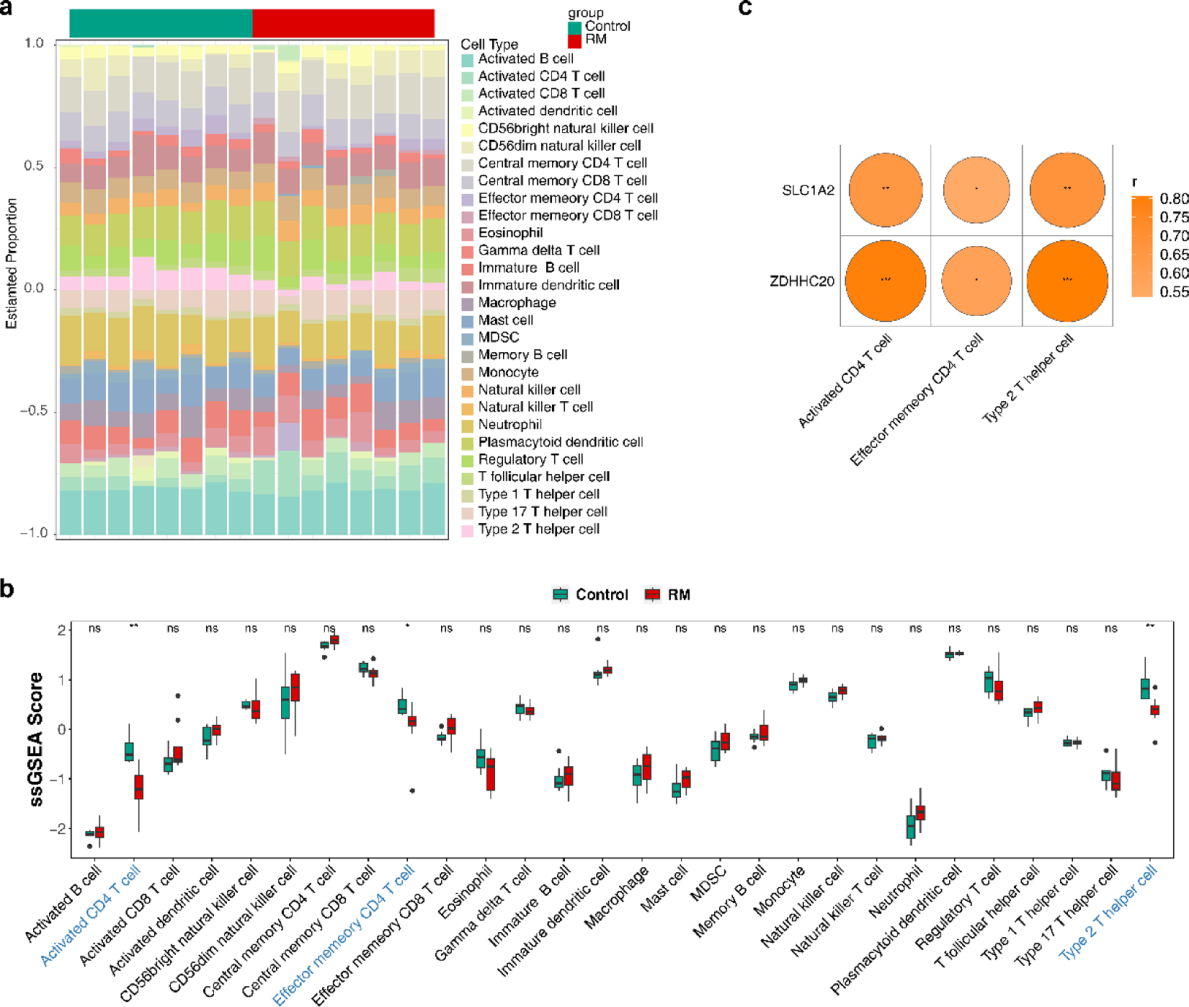
Evaluation of immune cell states in the control group and RM group using the ssGSEA method. (**a**) Stacked bar chart depicting the relative infiltration levels of 28 immune cell types across the RM and control groups. (**b**) Comparison of the infiltration levels of 28 immune cell types between the control and RM groups based on ssGSEA scores. Green bars represented the control group, and red bars represented the RM group. Significance levels in the figure were based on FDR (Benjamini-Hochberg)-adjusted p-values (Wilcoxon rank-sum test): * adjusted *p* < 0.05, ** adjusted *p* < 0.01; ns indicated adjusted *p* ≥ 0.05 (not statistically significant). Among these, effector memory CD4 + T cells showed a significant difference at the nominal level (raw *p* < 0.05) but did not remain significant after FDR adjustment (adjusted *p* > 0.05). (**c**) Visualization of the correlation between key genes SLC1A2 and ZDHHC20 and differentially infiltrating immune cell types. Orange indicates a positive correlation (significance *,*p* < 0.05; **, *p* < 0.01; ***, *p* < 0.001). The Spearman correlation coefficient represents the correlation between immune cells and key genes. In Fig. 4b, the statistical method for comparing immune cell infiltration levels between the RM and control groups is the Wilcoxon rank-sum test, with adjusted p-value < 0.05 defined as statistically significant.

To explore immune cell distributions between RM and control cohorts, expression data from the sequencing datasets were analyzed using ssGSEA (Fig. 4a, Table S8). The dominant immune cell populations comprised central memory CD4 T cells, plasmacytoid dendritic cells, neutrophils, and activated B cells. Comparative analysis revealed significant reductions in activated CD4 T cells and type 2 T helper cells in the RM group relative to controls (adjusted *p* < 0.05) (Fig. 4b). Correlation assessment indicated strong positive associations between the two identified key genes and these immune cell subsets. Specifically, ZDHHC20 exhibited the highest correlations with activated CD4 T cells (cor = 0.66, p < 0.05) and type 2 T helper cells (cor = 0.68, adjusted *p* < 0.05). SLC1A2 also showed notable positive correlations with activated CD4 T cells (cor = 0.79, adjusted *p* < 0.05) and type 2 T helper cells (cor = 0.81, adjusted *p* < 0.05) (Fig. 4c).

### Construction of the ceRNA network of key genes

**Fig. 5:**
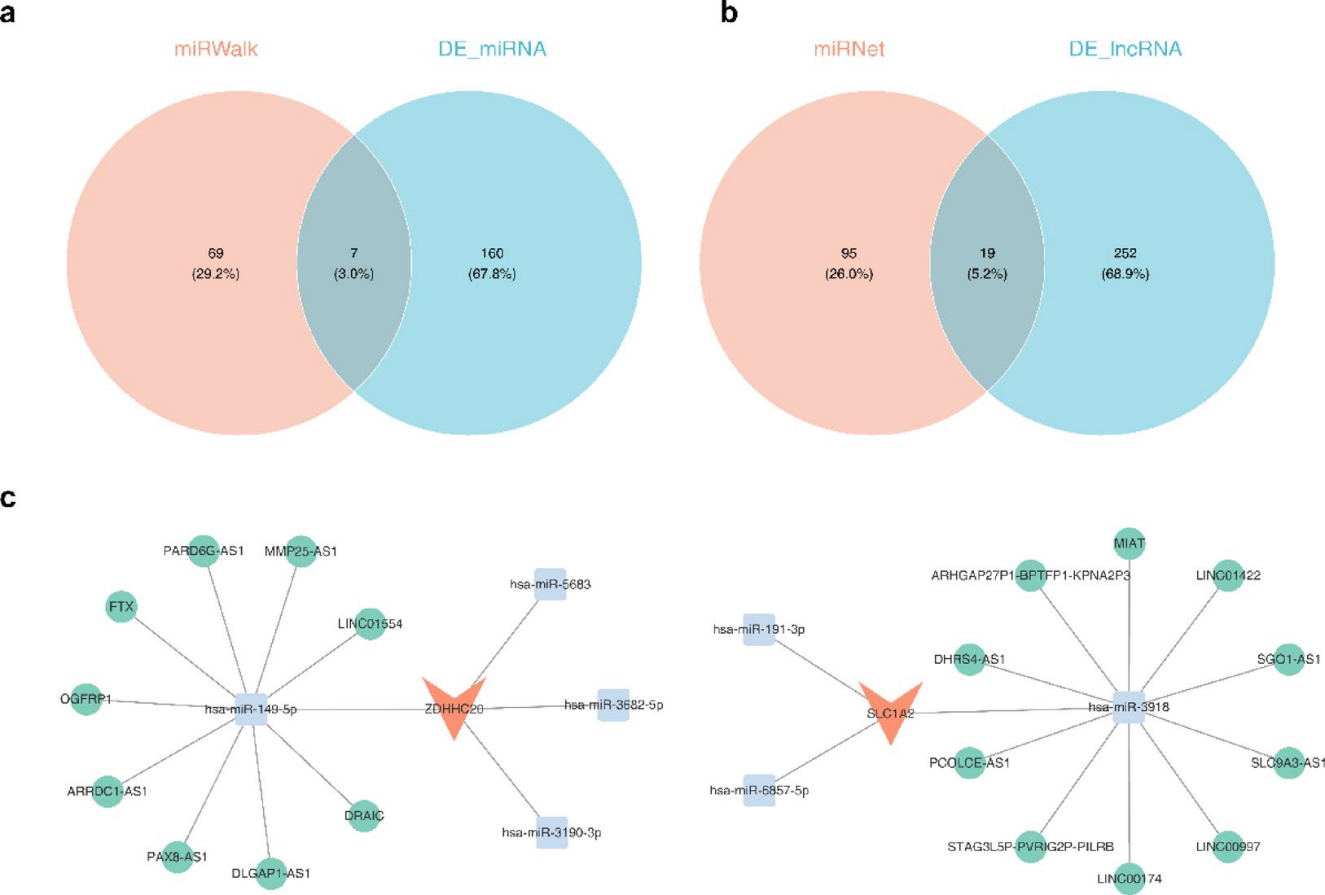
Construction of the ceRNA network of key genes. (**a**–**b**) Venn diagrams illustrating the overlap of mRNA–miRNA and miRNA–lncRNA interaction datasets. (**c**) Network visualization of regulatory relationships among genes (ZDHHC20 and SLC1A2) and associated molecules, including miRNAs and lncRNAs (orange inverted triangles represent key genes, blue squares indicate miRNAs, and green circles denote lncRNAs).

Based on the mRNA–miRNA and miRNA–lncRNA interaction data obtained from the miRWalk and miRNet databases, combined with DE-miRNA and DE-lncRNA profiles from sequencing datasets (Fig. 5a–b), a ceRNA regulatory network was constructed for the identified key genes. The analysis indicated that SLC1A2 and ZDHHC20 were modulated by seven miRNAs and nineteen lncRNAs (Fig. 5c). Specifically, SLC1A2 expression was regulated by hsa-miR-191-3p, hsa-miR-3918, and hsa-miR-6857-5p, while hsa-miR-3918 was in turn controlled by ten lncRNAs, including SGO1-AS1, SLC9A3-AS1, and LINC00997. ZDHHC20 was targeted by hsa-miR-5683, hsa-miR-149-5p, hsa-miR-3190-3p, and hsa-miR-3682-5p, among which hsa-miR-149-5p was influenced by nine lncRNAs such as PAX8-AS1, LINC01554, and ARRDC1-AS1. Analysis of this ceRNA regulatory network may enhance understanding of RM pathogenesis and contribute to potential diagnostic and therapeutic strategies, given the regulatory functions of miRNAs and lncRNAs in gene expression and disease development.

### Validation of the key genes

**Fig. 6:**
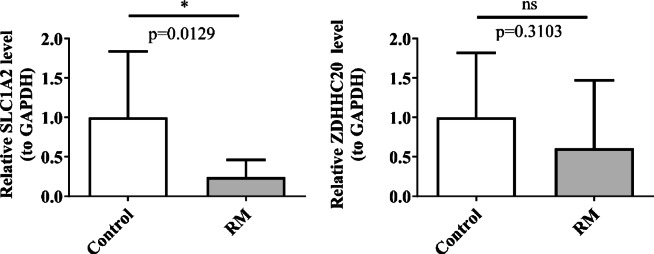
The analyses of qRT-PCR of SLC1A2 and ZDHHC20 between RM and control group. Expression levels of SLC1A2 and ZDHHC20 (x-axis: Control and RM groups; y-axis: relative expression normalized to GAPDH; error bars represent standard deviation (SD)) (Significance: ns, not significant; *, *p* < 0.05; **, *p* < 0.01; ***, *p* < 0.001).

Quantitative real-time polymerase chain reaction (qRT-PCR) was conducted on an additional ten pairs of clinical specimens to verify the expression of SLC1A2 and ZDHHC20. No significant differences were observed in patient age or gestational weeks between the RM and control groups. The qRT-PCR results demonstrated increased expression of both SLC1A2 and ZDHHC20 in the RM group relative to normal controls; however, only the upregulation of SLC1A2 reached statistical significance, whereas the difference in ZDHHC20 expression was not significant (Fig. 6).

## Discussion

RM constitutes a multifactorial clinical condition, with its etiological basis remaining undefined in a proportion of cases^38^. Aberrant DNA methylation may alter the expression of genes essential for embryonic development, whereas ciliary dysfunction can disrupt embryonic morphogenesis by impairing ciliary structure or function^39,40^. Further elucidation of the molecular and cellular interplay between these mechanisms is essential for advancing precise diagnostic and therapeutic strategies for this heterogeneous disorder. This investigation systematically analyzed the molecular landscape of RM through the integration of transcriptomic and DNA methylation data. SLC1A2 and ZDHHC20 were identified as methylation-regulated genes associated with ciliary function. The results broaden the current understanding of RM pathogenesis and reveal potential biomarkers and therapeutic targets for this reproductive disorder through functional enrichment analysis, immune landscape characterization, and experimental validation. Integration of LASSO and SVM-RFE algorithms further defined SLC1A2 and ZDHHC20 as central genes involved in RM.

The SLC1A2 (EAAT2) gene, located on chromosome 11p13, encodes a high-affinity, sodium-dependent glutamate transporter responsible for cellular uptake of L-glutamate and D/L-aspartate through Na + /H + cotransport and K + counter-transport mechanisms^41,42^. Predominantly expressed in astrocytes and neuronal terminals, SLC1A2 maintains synaptic glutamate equilibrium and protects neurons from excitotoxic injury by rapidly removing extracellular glutamate^43,44^. Excitatory amino acid transporters (EAATs), key modulators of glutamate signaling, have been implicated in autoimmune disorders affecting both the central nervous system and peripheral tissues^45^. Gan et al.^46^ demonstrated that lipopolysaccharide (LPS) induced Slc1a2-encoded EAAT2 expression through NF-κB activation, promoting pro-inflammatory macrophage polarization via mTORC1 signaling. The EAAT2–mTORC1 pathway thereby identifies macrophages as critical effectors in pathological processes such as sepsis^47^ and metabolic syndrome^48^. The involvement of Slc1a2 in RM has not been documented. Considering Yang et al.^49^ reported that glutamine/α-ketoglutarate imbalance in dNK cells contributed to pregnancy loss, and given SLC1A2’s established regulatory role in inflammatory macrophage polarization and metabolic reprogramming, a potential mechanistic connection to RM pathogenesis is plausible.

ZDHHC20, a member of the DHHC acyltransferase family, regulates multiple oncogenic processes through substrate-specific S-palmitoylation. This modification stabilizes key effector proteins such as GPX4 (ferroptosis resistance)^50^, FASN (lipogenesis)^51^, and AKT (proliferation signaling)^52^, thereby promoting metabolic reprogramming and therapeutic resistance in hepatocellular carcinoma. Beyond its metabolic role, ZDHHC20 reinforces MYC-driven pancreatic tumorigenesis by palmitoylating YTHDF3 to sustain oncogenic mRNA stability and modulates immune responses through lipid raft–associated targeting of ORAI1 (Ca^2+^ signaling) and CD80 (T-cell costimulation)^53^. Depletion of ZDHHC20 sensitizes cancer cells to EGFR inhibitors and ferroptosis inducers, establishing it as a multifunctional therapeutic candidate^54^. Collectively, the diverse actions of ZDHHC20 link tumor adaptability, immune evasion, and treatment resistance, emphasizing its mechanistic and clinical significance in precision oncology. Elevated ZDHHC20 expression was observed in RM tissues relative to controls; however, the absence of statistical significance in qPCR validation necessitates cautious interpretation. This divergence likely reflects sample size constraints or cellular heterogeneity inherent to placental and decidual tissues during early gestation. Moreover, transcriptional levels may not accurately reflect enzymatic activity, as ZDHHC20 function relies on post-translational regulation (e.g., autopalmitoylation) and substrate availability. Despite these limitations, its conserved involvement in immune regulation through CD80 palmitoylation and in oxidative stress defense via GPX4 stabilization corresponds with RM-associated immune dysregulation and oxidative imbalance, suggesting a context-dependent yet biologically plausible role for ZDHHC20 in maintaining pregnancy homeostasis.

In this study, functional enrichment analysis of DEGs identified key pathways and biological processes associated with RM, reflecting molecular mechanisms shared with other pathological conditions. The enriched pathways primarily involve three major domains. First, amino acid metabolism and neurotransmitter regulation: GO analysis revealed significant enrichment of genes related to synaptic transmission and glutamate/GABA-glutamine cycling (e.g., SLC1A2), consistent with observations in sleep deprivation models where reduced SLC1A2 expression disrupts astrocyte–neuron metabolic interactions, leading to cognitive dysfunction^55^. Such results indicate a conserved function of amino acid transporters in sustaining cellular equilibrium under stress. Moreover, SLC1A2’s role in ferroptosis, driven by glutathione depletion, further connects its dysregulation to oxidative stress in RM^52^. Second, immune and inflammatory pathways: KEGG analysis identified enrichment in cytokine production and leukocyte-mediated immune responses, consistent with immune perturbations observed in RM. Decreased Th2 cell infiltration parallels mechanisms in sepsis, where SLC1A2 overexpression enhances pro-inflammatory macrophage polarization through mTORC1 activation^51,56^, indicating an overlapping immune–metabolic regulatory axis across inflammatory disorders. Third, protein localization and stability: Enrichment of pathways such as protein targeting to vacuoles and ubiquitin-mediated proteolysis corresponds with research on ZDHHC20, demonstrating that palmitoylation stabilizes oncoproteins (e.g., FASN, MYC) by competing with ubiquitination, thereby promoting tumor progression^51,57^. Similarly, aberrant regulation of protein stability in RM may disrupt trophoblast invasion and placental formation.

PPI network analysis of the 14 candidate genes revealed the molecular connectivity underlying RM. The network exhibited a relatively simple topology, comprising only four interaction pairs, with HSPA4 identified as a central node interacting with several genes, including DNAJC8 (co-chaperone), TSG101 (ESCRT-I component), and SLC1A2 (glutamate transporter). As a member of the HSP70 chaperone family, HSPA4 silencing markedly suppresses cell proliferation and induces ferroptosis^58^, indicating its involvement in stress adaptation and protein homeostasis. Characterizing these molecular associations may clarify the functional interdependence among genes and support the identification of potential therapeutic targets. Furthermore, the observed HSPA4–SLC1A2 linkage implies a stabilizing chaperone function of HSPA4 toward SLC1A2, whose glutamate transport activity is essential for maintaining redox equilibrium. In glioblastoma, HSP70 chaperones preserve SLC1A2 stability, sustaining cystine uptake and glutathione biosynthesis to mitigate ferroptosis^59^. By analogy, HSPA4 may protect trophoblasts from oxidative stress in RM.

The GSEA analysis provided a system-level insight into dysregulated biological pathways and molecular networks associated with RM. SLC1A2 primarily participated in two interrelated pathways, with significant enrichment of oxidative phosphorylation (OXPHOS) and ferroptosis observed in SLC1A2-high RM samples. This enrichment is consistent with SLC1A2’s function in maintaining cellular redox equilibrium through glutamate transport and glutathione biosynthesis. In glioblastoma, elevated SLC1A2 expression enhances OXPHOS to counteract ferroptosis through mitochondrial complex I activity^60^. Within RM, disrupted OXPHOS may impair trophoblast energy metabolism, aggravating placental oxidative stress—a phenomenon similarly documented in preeclampsia^61^, where mitochondrial dysfunction correlates with disease progression. Additionally, the enrichment of SLE-associated genes (e.g., IFIT3, ISG15) indicates a potential autoimmune link between RM and SLE^62^. These findings imply that SLC1A2-driven modulation of decidual macrophage polarization via mTORC1 signaling may converge with autoimmune mechanisms, forming a molecular interface between metabolic imbalance and immune dysregulation.

Enrichment analysis associated with ZDHHC20 revealed upregulation of ubiquitin-mediated proteolysis and cell cycle pathways, reflecting its dual regulatory functions in protein homeostasis and cellular proliferation. In hepatocellular carcinoma (HCC), ZDHHC20 promotes AKT stabilization through palmitoylation, thereby enhancing cell cycle progression and conferring chemoresistance^52^. Similarly, in RM, ZDHHC20-dependent palmitoylation of FASN or GPX4 may interfere with protein degradation dynamics, resulting in aberrant trophoblast proliferation or maladaptive oxidative stress responses.

Our ssGSEA analysis revealed diminished infiltration of Th2 cells and effector memory CD4 + T cells in RM tissues, consistent with previous observations^63^. Th2-derived cytokines (e.g., IL-4, IL-10) support trophoblast viability and angiogenesis, whereas Treg cells maintain immune tolerance through CTLA-4 and TGF-β signaling^63^. The positive association between ZDHHC20 and Th2 cells suggests that ZDHHC20-mediated palmitoylation of costimulatory molecules such as CD80 may stabilize immune synapse assembly, a mechanism recently linked to T cell exhaustion during chronic viral infections. Conversely, Th2/Treg insufficiency in RM may result from SLC1A2-induced mTORC1 hyperactivation, driving macrophage polarization toward a pro-inflammatory M1 phenotype, as demonstrated in sepsis models^64^.

The ceRNA network centered on SLC1A2 and ZDHHC20 delineates a hierarchical post-transcriptional regulatory framework in RM. SLC1A2 is co-regulated by hsa-miR-3918 and hsa-miR-6857-5p alongside hsa-miR-191-3p, whereas ZDHHC20 is embedded in a network governed by upstream lncRNAs such as PAX8-AS1 and SGO1-AS1, which act as molecular sponges for competing miRNAs. These interactions form cascading regulatory loops; for instance, PAX8-AS1 sequesters hsa-miR-3918, thereby influencing SLC1A2 expression, reflecting the multilayered control of genes essential for embryonic development.

The ceRNA network thus represents a nexus of epigenetic dysregulation in RM, where concurrent perturbations of miRNAs and lncRNAs disturb transcriptional equilibrium. Therapeutic modulation of specific non-coding RNAs—such as restoring hsa-miR-149-5p to attenuate placental inflammation or adjusting PAX8-AS1 to normalize SLC1A2 expression—may provide targeted and mechanistically grounded interventions for this complex reproductive disorder.

This investigation integrates multi-omics datasets (transcriptomic, methylomic, and non-coding RNA networks) with machine learning approaches to dissect the epigenetic and metabolic mechanisms underlying recurrent miscarriage (RM)—a strategy rarely employed in previous RM studies, which have primarily focused on single-omics analysis or candidate gene validation^65^. To enhance the robustness of biomarker identification, we combined LASSO (for dimensionality reduction and overfitting prevention) and SVM-RFE (for recursive feature selection) algorithms, a two-step validation framework that minimizes false positives and ensures the identified core genes (SLC1A2 and ZDHHC20) are both methylation-associated and cilia-related.

Furthermore, we employed single-sample gene set enrichment analysis (ssGSEA) to quantify immune cell infiltration patterns, which were then correlated with core gene expression to link molecular signatures with immune dysregulation—a key pathogenic feature of RM. Experimental validation via qRT-PCR (n = 8 per group) confirmed the differential expression of these genes in an independent subset, reinforcing their potential translational value. The construction of a ceRNA regulatory network (connecting core genes with miRNAs and lncRNAs) further delineates a multi-layered regulatory landscape, offering novel insights into how epigenetic and post-transcriptional modifications coordinate to influence RM pathogenesis.

Despite these strengths, several limitations should be acknowledged. First, the clinical validation cohort (n = 10 pairs for qRT-PCR) is relatively small, which may reduce statistical power to detect subtle expression changes, particularly for ZDHHC20. However, this sample size is comparable to similar exploratory studies in RM^65^, and the consistency between multi-omics screening and qRT-PCR results supports the reliability of our findings. Future studies with larger cohorts (n > 50) are warranted to confirm these associations.

Second, the use of bulk RNA-seq data may mask cell-type-specific transcriptional patterns in placental or immune cells (e.g., decidual macrophages). To partially address this, we inferred immune cell proportions via ssGSEA, which revealed correlations between core genes and infiltrating immune cells, providing indirect evidence for cell-type-specific regulation. Single-cell RNA sequencing of placental or decidual tissues will be critical to clarify the precise cellular sources of these transcriptional changes.

Third, the proposed roles of SLC1A2 and ZDHHC20 in ciliary function, ferroptosis, and immune modulation are based on bioinformatic predictions and correlation analyses. Targeted in vitro experiments (e.g., cilia formation assays in trophoblast cells with SLC1A2 knockdown) and in vivo models (e.g., ZDHHC20 knockout mice) are needed to verify their mechanistic contributions.

Finally, our analysis focused on DNA methylation and non-coding RNAs, excluding other epigenetic layers (e.g., histone modifications, chromatin accessibility) and proteomic validation. Integrating ChIP-seq or ATAC-seq data, along with western blotting or mass spectrometry, will help resolve post-transcriptional and translational regulatory mechanisms in future work.

## Conclusion

This multi-omics investigation comprehensively characterizes the molecular framework of RM by integrating transcriptomic, epigenetic, and immune microenvironmental analyses to identify novel determinants of disease development. Through stringent bioinformatic screening combined with machine learning algorithms, SLC1A2 and ZDHHC20 were defined as methylation-regulated, cilia-associated hub genes implicated in ferroptosis, ubiquitin-mediated proteolysis, and immune dysregulation.

By linking epigenetic modulation, metabolic imbalance, and immune perturbation, this study reframes RM as a biologically interpretable disorder rather than an unexplained clinical condition. The identified molecular signatures enrich current understanding of RM pathophysiology and establish a framework for exploring related pregnancy disorders involving placental dysfunction. Therapeutic strategies directed at these key regulatory nodes—using small-molecule agents such as ferroptosis inhibitors or miRNA-targeted approaches—may enable precision-based interventions to enhance pregnancy outcomes.

## Supplementary Information

Below is the link to the electronic supplementary material.

Supplementary Material 1

Supplementary Material 2

Supplementary Material 3

Supplementary Material 4

Supplementary Material 5

Supplementary Material 6

Supplementary Material 7

Supplementary Material 8

Supplementary Material 9

Supplementary Material 10

Supplementary Material 11

Supplementary Material 12

## Data Availability

The raw human RNA-seq sequence data are available under BioProject accession number PRJNA1271476 in the NCBI BioProject database (https://www.ncbi.nlm.nih.gov/bioproject/) and other data that support the findings of this study are available from the corresponding author upon request.
