# Supplementary figures and images for: Integration of transcriptome and DNA methylation reveals the mechanism of cilia-related genes in recurrent miscarriage
Source: Sci Rep. 2026 May 9;16:21324. doi: 10.1038/s41598-026-52154-x (PMC13346893; doi:10.1038/s41598-026-52154-x)

DE-miRNAs\_volcano

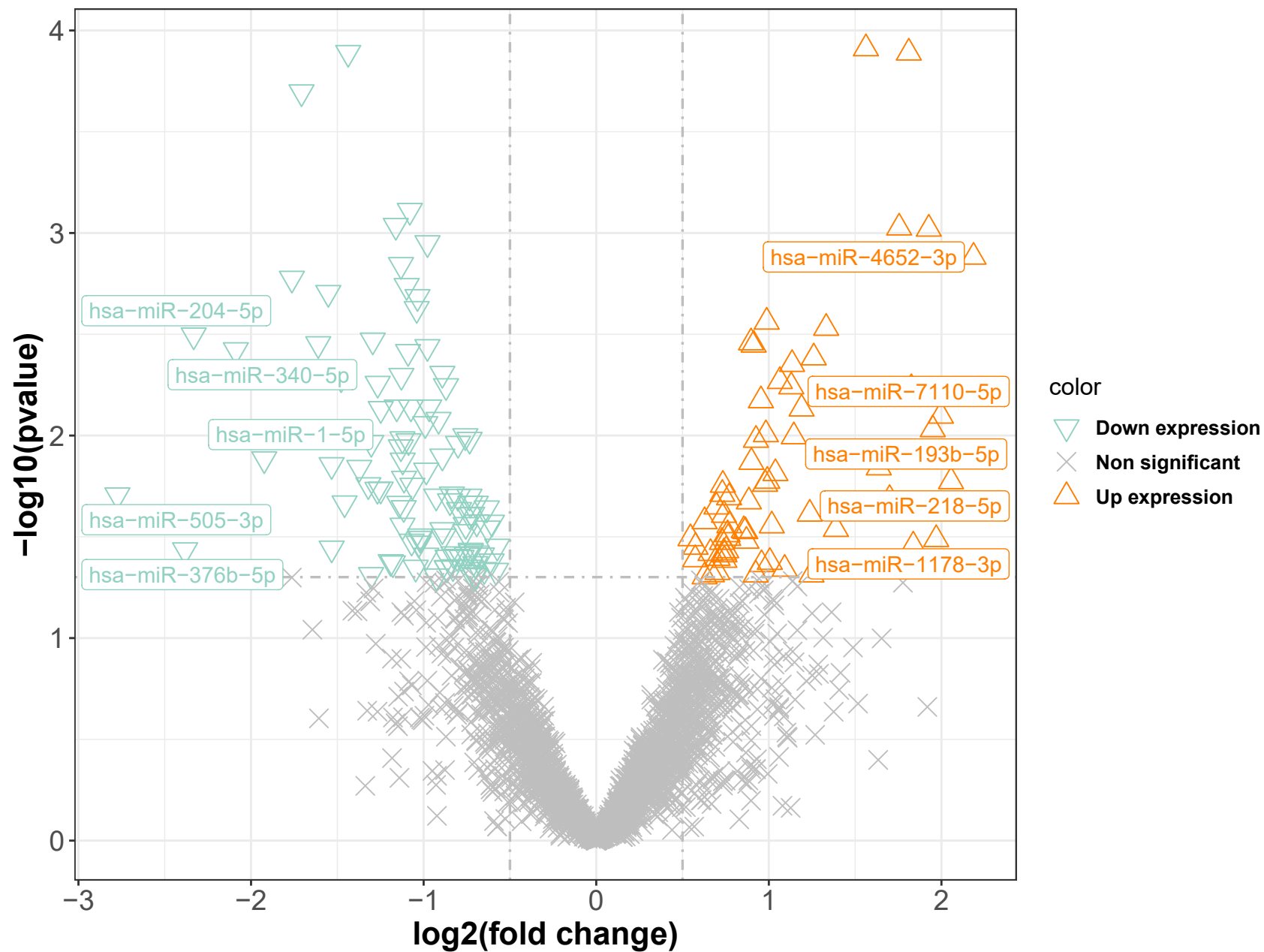

Supplement: Supplementary file 1 — Supplementary Material 1 [file 41598_2026_52154_MOESM1_ESM.pdf]

DE-lncRNAs\_volcano

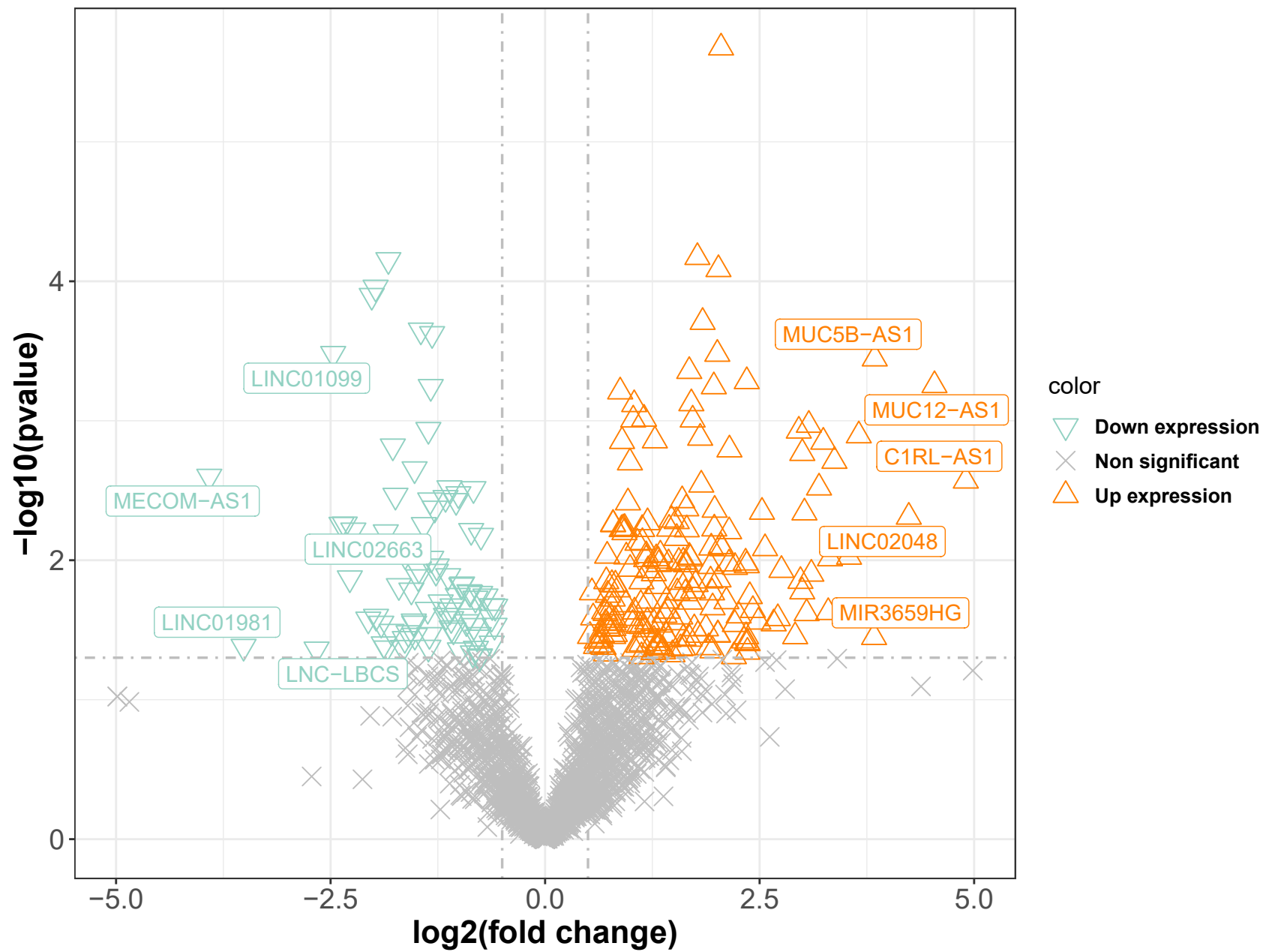

Supplement: Supplementary file 2 — Supplementary Material 2 [file 41598_2026_52154_MOESM2_ESM.pdf]

DE-miRNA Distribution as heatmap

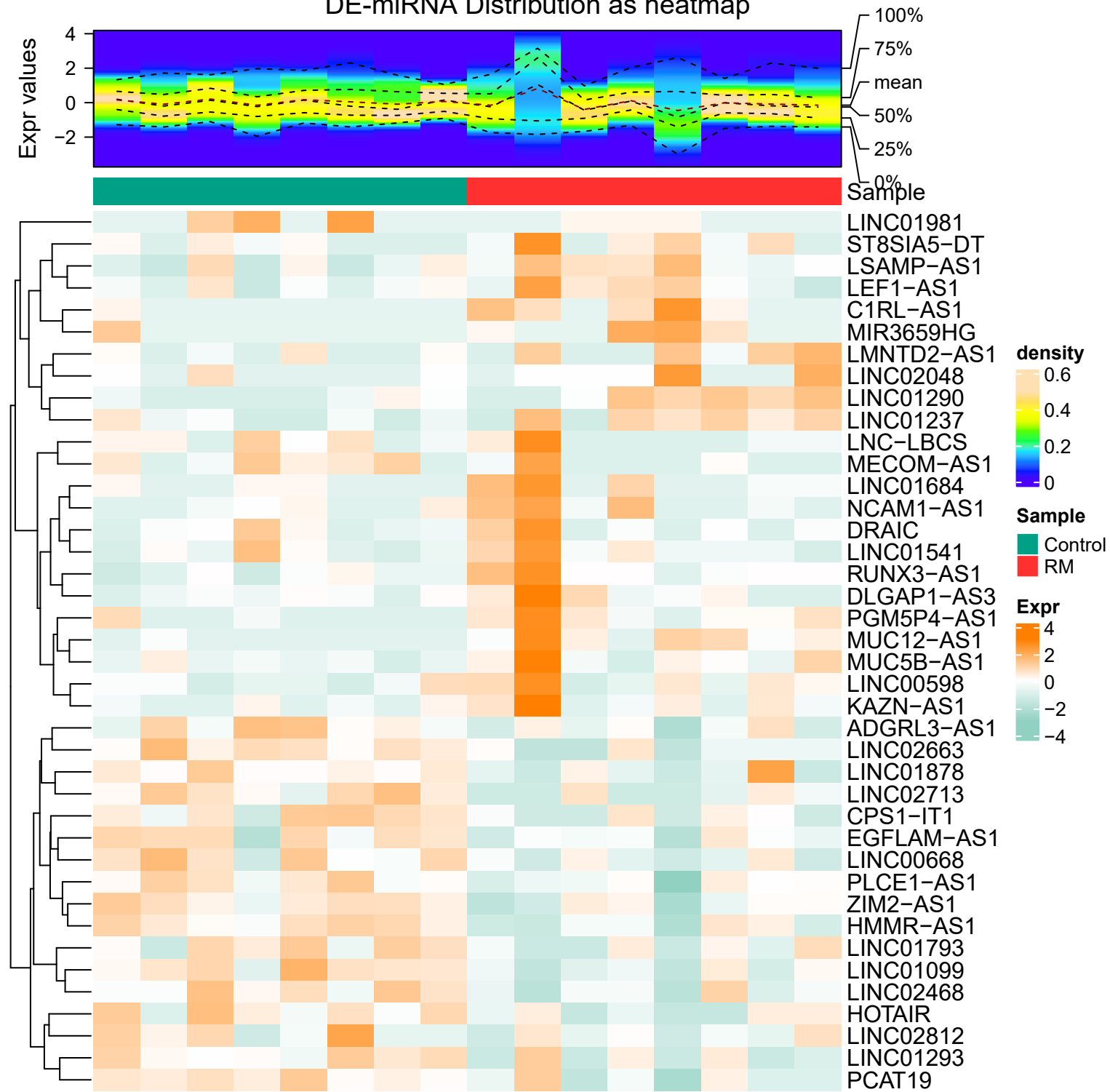

Supplement: Supplementary file 3 — Supplementary Material 3 [file 41598_2026_52154_MOESM3_ESM.pdf]

DE-miRNAs Distribution as heatmap

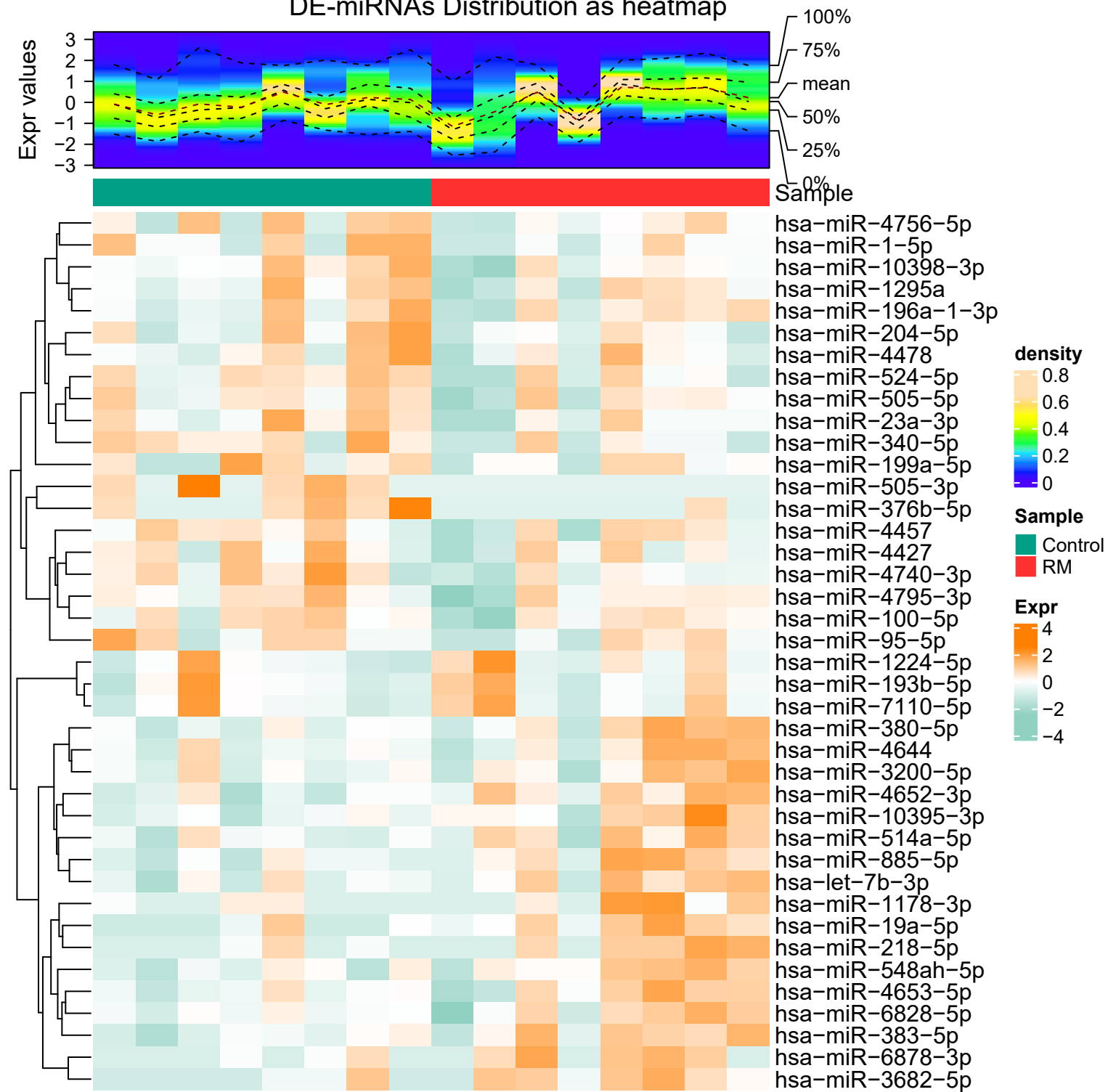

Supplement: Supplementary file 4 — Supplementary Material 4 [file 41598_2026_52154_MOESM4_ESM.pdf]
