# Supplementary material for: Integration of transcriptome and DNA methylation reveals the mechanism of cilia-related genes in recurrent miscarriage
Source: Sci Rep. 2026 May 9;16:21324. doi: 10.1038/s41598-026-52154-x (PMC13346893; doi:10.1038/s41598-026-52154-x)
Supplement: Supplementary file 6 — Supplementary Material 6 [file 41598_2026_52154_MOESM6_ESM.docx]

Table S1 Clinical sample information for qRT-PCR validation (n = 10 pairs).

| Variables | Control |  | RM | Raw P-value | Adjusted p-value |
| --- | --- | --- | --- | --- | --- |
|  | (n = 10) |  | (n = 10) |  |  |
| Age, y | 33.10±4.80 |  | 33.90±3.90 | 0.726 | 0.726 |
| BMI, kg/m^2^ | 20.07±1.41 |  | 20.70±1.50 | 0.589 | 0.884 |
| Gestational age, week | 6.80±0.70 |  | 7.00±0.67 | 0.063 | 0.189 |
