# Supplementary material for: Integration of transcriptome and DNA methylation reveals the mechanism of cilia-related genes in recurrent miscarriage
Source: Sci Rep. 2026 May 9;16:21324. doi: 10.1038/s41598-026-52154-x (PMC13346893; doi:10.1038/s41598-026-52154-x)
Supplement: Supplementary file 7 — Supplementary Material 7 [file 41598_2026_52154_MOESM7_ESM.docx]

Table S2 The primer sequences of biomarkers for quantitative real-time polymerase chain reaction (qRT-PCR).

| primer | sequence |
| --- | --- |
| SLC1A2 F | CTGGCCGAGAAGGATCTGGA |
| SLC1A2 R | CGTGAACTCGTAGCGCGAA |
| ZDHHC20 F | ACCCGCTGTTCTCTCTACCT |
| ZDHHC20 R | CACACGGTGGGAATGCAATG |
